# Response to ‘Pulmonary arterial hypertension: An update’ by M. Correale (10.1007/s12471-012-0245-2)

**DOI:** 10.1007/s12471-012-0247-0

**Published:** 2012-02-14

**Authors:** E. S. Hoendermis

**Affiliations:** Department of Cardiology, University Medical Centre Groningen, University of Groningen, PO Box 30.001, 9700 RB Groningen, the Netherlands

## Comment

Thank you for your comment. Indeed stress echocardiography is a promising tool for the evaluation of the phenomenon of exercise-induced pulmonary hypertension (PH). However, as you already mentioned, cut-off values and influence of other factors such as age and standardisation of exercise protocols are lacking and the predictive role of PH at exercise is not well understood. For these reasons the definition of exercise-induced PH, given by an invasively measured mean pulmonary artery pressure of more than 30 mmHg at exercise was withdrawn from the guidelines. Available data did not support this definition. Stress echocardiography as a noninvasive tool is more easily available but has limitations in estimating pulmonary pressure at rest and exercise compared with invasive measurements. Stress echocardiography, but also invasive studies, will play an important role to evaluate a good definition and the prognostic value of exercise-induced PH. At the moment both are not advisable as routine practice.

